# Combining fossil taxa with and without morphological data improves dated phylogenetic analyses

**DOI:** 10.1098/rsbl.2025.0205

**Published:** 2025-08-13

**Authors:** Mark C. Nikolic, Rachel C. M. Warnock, Melanie J. Hopkins

**Affiliations:** ^1^ Richard Gilder Graduate School, American Museum of Natural History, New York, NY, USA; ^2^ Division of Paleontology (Invertebrates), American Museum of Natural History, New York, NY, USA; ^3^ Department of Geography and Geosciences, Friedrich-Alexander-Universität Erlangen-Nürnberg GeoZentrum Nordbayern, Erlangen, Bavaria, Germany

**Keywords:** fossilized birth–death, phylogenetic inference, stratigraphic congruence, morphological data, divergence times, fossils

## Abstract

The fossilized birth–death (FBD) model has become an increasingly popular method for inferring dated phylogenies. It is especially useful for incorporating fossil data into such analyses, integrating fossils along with their age information directly into the tree as tips or sampled ancestors. Two approaches are common for placing fossil taxa in trees: inference based on morphological character data or using taxonomic constraints to control their topological placement. These approaches have historically been treated as alternatives, and for phylogenetic inference of entirely extinct organisms, additional related fossil taxa other than those for which morphology is available are generally overlooked. Here, for the first time, we implement a combined approach on an empirical dataset for a group of trilobites. We use a morphological matrix and ages for 56 taxa and age information for another 196 taxa from the Paleobiology Database. To evaluate the effects of a combined approach, we conducted FBD-dated phylogenetic analyses using the combined dataset with morphology and taxonomic constraints and compared them to analyses of taxa with morphology alone. We find that a combined approach yields topologies that are more stratigraphically congruent, substantially more precise parameter estimates (e.g. divergence times) and more informative tree distributions. These findings are a consequence of the substantial increase in stratigraphic age information and a more representative sample of the temporal distributions of the group.

## 1. Introduction

Phylogenetic trees provide necessary context to the study of the evolution of organisms throughout Earth’s history. The fossil and stratigraphic record, in turn, provide the opportunity to directly incorporate the dimension of time in such studies and further inform phylogenies: a quality of this record recognized for at least half a century [1]. Fossils here can directly provide two sources of information: morphology and age. The development of the increasingly popular fossilized birth–death (FBD) process [2–4] has provided an especially useful model for incorporating fossil data and some of its idiosyncrasies into phylogenetic analyses (reviewed in [5]). The FBD process explicitly considers the lineage diversification (speciation and extinction) process and the fossil sampling/recovery process together, incorporating fossils directly into the tree, while accounting for uncertainty in age and phylogenetic placement in a Bayesian framework.

Fossils can be incorporated as tips into FBD analyses either with or without morphological character data along with their age. When character data are used, the topological placement of fossil taxa can be estimated based on these data with greater precision. Furthermore, combining fossil morphological data with morphological data for extant taxa, along with a molecular alignment, results in the so-called total-evidence analysis [6–9]. Alternatively, fossils without morphological data can be placed in the tree using topological constraints [10]. Usually, these constraints are informed by taxonomy, restricting certain clades to be monophyletic based on higher groupings (e.g. genus or family [11]). Taxonomic constraints thereby allow the age information of fossils to still be appropriately integrated into the analysis in the absence of morphological data. The former strategy has been referred to as the ‘resolved’ FBD model and the latter the ‘unresolved’ FBD model [12]. Regardless of the strategy used, the desirable, net-positive effects of fossils (and stratigraphic age information) in phylogenetic analyses are apparent and strongly supported [13–15]).

Morphological data and taxonomic constraints (resolved versus unresolved) have historically been treated as alternative strategies [10,12], and in practice have been used as such. While the FBD model has been successfully utilized for phylogenetic analysis of entirely extinct groups [16–21], only the resolved FBD model has been used in this context. The most apparent reason for this is, given the lack of molecular data for fossils, morphological data are needed to infer tree topologies. Fossil taxa are therefore sampled to have the most abundant or diagnostic morphological characters, even if those taxa have a temporal distribution that is appreciably different from the overall distribution of known occurrences for that group. This violates the FBD model assumption of uniform/random sampling and in some cases will provide a biased approximation of the true fossil sampling process [12], which can in turn bias the output of inference using the FBD model.

A combination of the two approaches (morphological data and taxonomic constraints) is theoretically possible. Simulations have indeed shown that a combined approach produces more accurate parameter estimates than either strategy alone [10]. Furthermore, because the distribution of fossil sampling times informs the FBD model parameters—much like other quantitative-palaeobiology methods used to infer speciation and extinction rates (like the three-timer [22] or boundary-crosser rates [23,24])—more fossil data (even as occurrences) are expected to increase precision in divergence times and diversification rate parameters. Occurrence and age information can even influence inferred tree topologies [25] and appear to hold vital phylogenetic information [14]. Therefore, using only fossil taxa with abundant morphological characters and ignoring the rest underutilizes the information in the fossil record. Combined analyses would therefore allow researchers to take full advantage of the fossil record, particularly for analyses of extinct groups.

Here we implement a combined approach, FBD phylogenetic analysis on an empirical dataset for a group of trilobites (morphological matrix and ages) by including stratigraphic age information from occurrences for other members of the group from the Paleobiology Database (PBDB; https://paleobiodb.org). We refer to the combined analysis as a ‘semi-resolved’ analysis and compare it to a ‘resolved’ analysis, where we only include taxa with morphological data coded into the morphological matrix. In doing so, we test the effect of both a substantial increase in stratigraphic age information and a more realistic fossil sampling distribution on FBD analyses in an empirical setting. We explore these effects by conducting a thorough examination of the posterior distributions of inferred divergence times and trees. To compare the quality of topologies when the true tree is unknown, we assess the stratigraphic congruence of trees produced from each analysis. Finally, we explore leaf stability and the quality of consensus trees by assessing how informative of the posterior distribution they are.

## 2. Methods

We conducted both resolved and semi-resolved tip-dated FBD phylogenetic analyses using the morphological character matrix for a phylogenetic analysis and revision of the order Aulacopleurida, which is available on MorphoBank [26]. This matrix consists of 254 characters for 56 species, spanning approx. 125 ma, from the middle Cambrian to the Middle Devonian (the largest number of characters in a trilobite character matrix to date). Age data for the taxa in this morphological matrix were (mostly) obtained as high-resolution biozone intervals from the literature. Biozonation information was not available for some select species and so formation or regional stage intervals were used. These intervals were then correlated to a global scale based on Gradstein *et al*. [27] to obtain absolute age intervals.

For the semi-resolved analysis, we retrieved all occurrences for every genus in the morphological matrix from the PBDB (electronic supplementary material, figure S1A). We cleaned this dataset by removing taxa that were not identified to species and/or were associated with very imprecise stratigraphic intervals (e.g. the entire Cambrian). We also removed species that were in the morphological dataset because we already had higher-resolution temporal information from the literature. Following the cleaning procedure, we had a dataset with age information for 194 more species and a combined dataset of 250 species. This dataset then covered the entire range of our lineage of interest with stratigraphic gaps minimized (electronic supplementary material, figure S1B) compared to the temporal distribution of species in the morphological matrix alone (electronic supplementary material, figure S1C). For the PBDB dataset, we then randomly subsampled an occurrence for each species to provide the age interval for that species.

We ran all analyses in the software Beast 2.6.7 [28] with the constant rates FBD model as implemented in the sampled ancestors (SA) package [2]. We ran the ‘resolved’ analyses for 1 000 000 000 MCMC generations and the ‘semi-resolved’ analyses for 2 000 000 000 generations. All analyses had the same priors and parameters, except for the origin time prior. Because of the conflicting hypotheses of the trilobite origin time [29,30], we tested the influence of both a uniform and exponential prior on both resolved and semi-resolved analyses (electronic supplementary material, table S1). The exponential prior supports a stricter reading of the fossil record by assigning higher origin probabilities closer to the first (oldest) fossil occurrence. We restricted the phylogenetic placement of the 194 taxa without morphology by using genus-level monophyletic clade constraints. Since all genera in our tree are represented in our morphological matrix, all species could be placed within a monophyletic generic lineage with at least one member of that genus having morphological information.

To make direct and computationally tractable comparisons, we focused the analyses of posterior tree distributions on the output from the resolved and semi-resolved analyses with the exponential origin prior, as this prior choice had minimal effect. We first pruned the 194 species without morphology from the semi-resolved analysis trees, so that trees from both analyses had the same leafset, then randomly subsampled the distributions to 900 trees each (1% of the post-burnin tree sample). We calculated stratigraphic congruence metrics for each tree in each posterior distribution using the R package ‘strap’ [31]. We used the stratigraphic consistency index (SCI [32]), minimum implied gap (MIG [33]) and gap excess ratio (GER [34]).

To visualize the effects on topology and, consequently, stratigraphic congruence, we also produced a treespace of these trees from both posterior distributions using the R package ‘TreeDist’ [35][36]. Furthermore, we used the treespace to produce a stratigraphic congruence ‘landscape’. We also explored leaf stability through the effect of ‘rogue’ taxa using the ‘Rogue’ R package [37]. Finally, we assessed the quality of consensus trees by calculating the amount of information (bits) they communicated about clades in the posterior distribution using the splitwise phylogenetic information content (SPIC) [37].

A detailed explanation of the dataset, analytical set-ups, convergence assessment and analysis of posterior distributions and stratigraphic congruence is provided in electronic supplementary material, Supplementary Methods. R code for writing Beast XML files with large amounts of PDBD information and taxonomic constraints, and all analyses of the posterior tree distribution, are available in the electronic supplementary material.

## 3. Results

### (a) Stratigraphic congruence and topology

**Figure 1.**
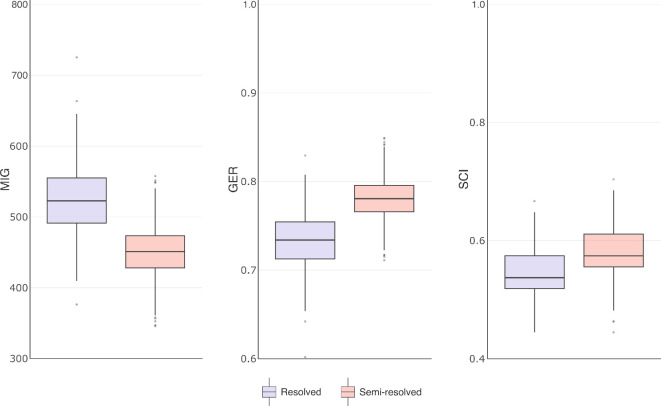
Stratigraphic congruence metrics for the posterior distributions of trees produced from resolved and semi-resolved FBD analyses. MIG = minimum implied gap (lower is better), GER = gap excess ratio (higher is better), SCI = stratigraphic consistency index (higher is better). All pairwise *p* values <0.05.

**Figure 2.**
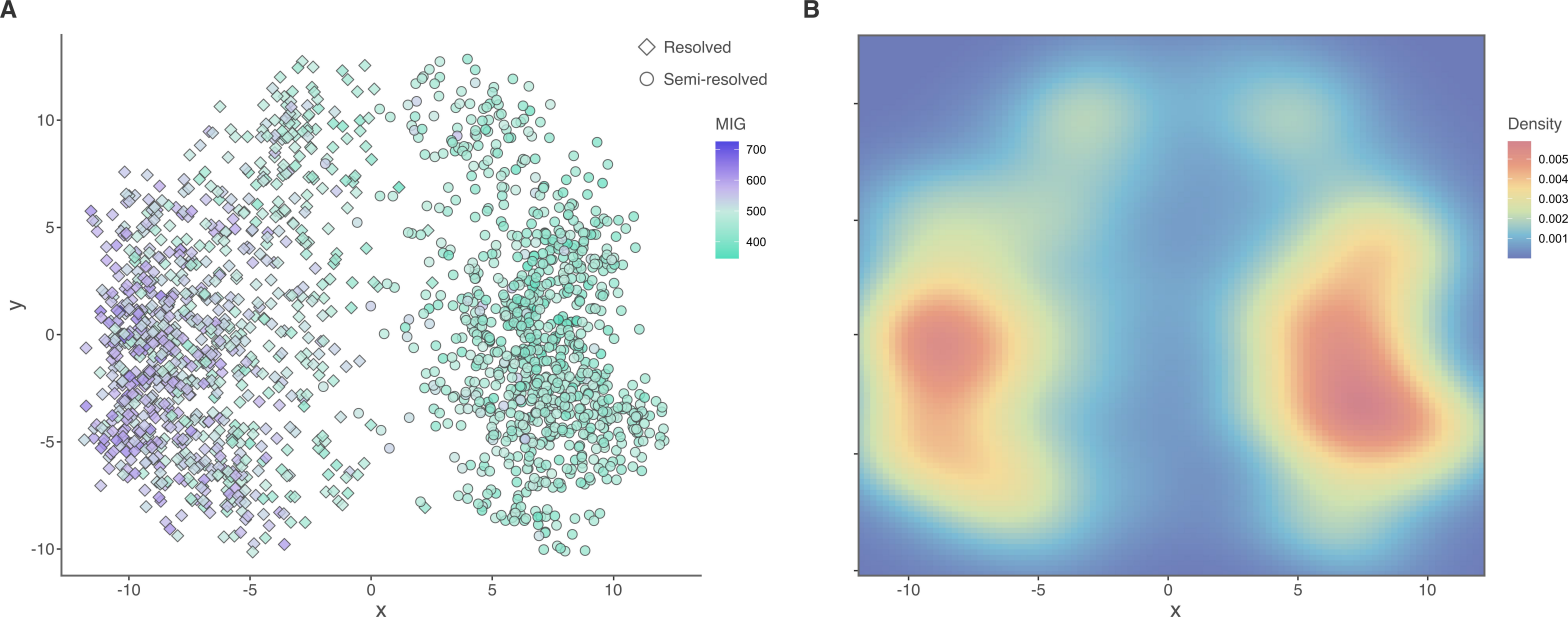
Treespace produced from subsampled trees from both analysis types. (A) Stratigraphic congruence ‘landscape’ (points coloured by MIG; lower values indicate higher stratigraphic congruence). Analysis type is represented by point shape. (B) Heat map of density in the same treespace. Warmer colours indicate a higher density of trees clustering in that region of treespace.

The semi-resolved FBD analysis was superior in all measures. Semi-resolved analyses produced trees that were significantly and substantially more stratigraphically congruent under all metrics (figure 1): a consequence of different topologies in the posterior tree distributions. The effect is more pronounced in the MIG and GER than the SCI, which is a consequence of the SCI ignoring implied gaps (i.e. ghost ranges). When analysed together, trees from each analysis largely occupy separate regions of treespace (at least in the first dimension). Consequently, they occupy distinct regions of the stratigraphic congruence tree ‘landscape’, with a clear gradient of congruence (figure 2A: MIG; electronic supplementary material, figure S2, SCI and GER).

There were important differences in the spread of each distribution in treespace. Trees from the semi-resolved analyses clustered more densely around the region of highest stratigraphic congruence in the landscape (figure 2B). Meanwhile, trees from the resolved analyses appeared largely on a local optimum of lower stratigraphic congruence and were also more diffuse. This is reinforced by the semi-resolved distribution having lower sum of variances, sum of ranges and mean centroid distance (electronic supplementary material, table S3). While topological differences are apparent in the first dimension of the treespace, the distributions overlap in other dimensions (electronic supplementary material, figure S3).

### (b) Consensus/summary trees and leaf stability

Node supports were relatively low for some parts of the tree in both analyses and the consensus trees for this group of trilobites are not highly resolved (electronic supplementary material, figure S4). However, key parts of the tree—such as those nodes defining the base of higher taxonomic groupings (superfamilies/suborders)—had higher support and were more confidently recovered in the semi-resolved analysis (electronic supplementary material, figures S4 and S5). Additionally, the sum of posterior probabilities for all nodes in the maximum clade credibility (MCC) tree from the semi-resolved analysis was higher than for the resolved analysis (table 1). Semi-resolved analyses also produced slightly more resolved majority rule consensus trees, having one more node than the resolved analysis consensus tree (table 1; electronic supplementary material, figure S4).

**Table 1.** Statistics related to consensus trees (majority rule and maximum clade credibility) for the posterior distributions produced from the resolved and semi-resolved FBD analyses. Statistics concern the majority rule consensus tree, except for the sum of posterior probabilities, which concerns the MCC tree.

analysis	N nodes	N rogues	information content	mean improvement from rogue removal	sum of posterior probabilities (MCC tree)
resolved	20	12	129.38	6.58	18.96
semi-resolved	21	6	185.59	5.49	21.68

The semi-resolved analysis consensus tree also had a higher information content (SPIC) than did the resolved analysis consensus tree (table 1). Higher information content means the semi-resolved analysis consensus captured deeper nodes/larger clade relationships, reflecting greater stability of those relationships in the posterior tree distribution.

Rogue taxa are those taxa whose phylogenetic position is particularly ‘unstable’ in a distribution of trees. These taxa create issues in summarizing tree distributions. Here, the resolved analysis had a posterior distribution with twice as many taxa displaying rogue behaviour than the semi-resolved analysis. Furthermore, each rogue in the resolved distribution was more detrimental to the consensus tree on average than were the semi-resolved consensus rogues. The removal of rogue taxa also had the effect of increasing the MIG in both analyses (electronic supplementary material, figure S6).

### (c) Divergence and origin times

**Figure 3.**
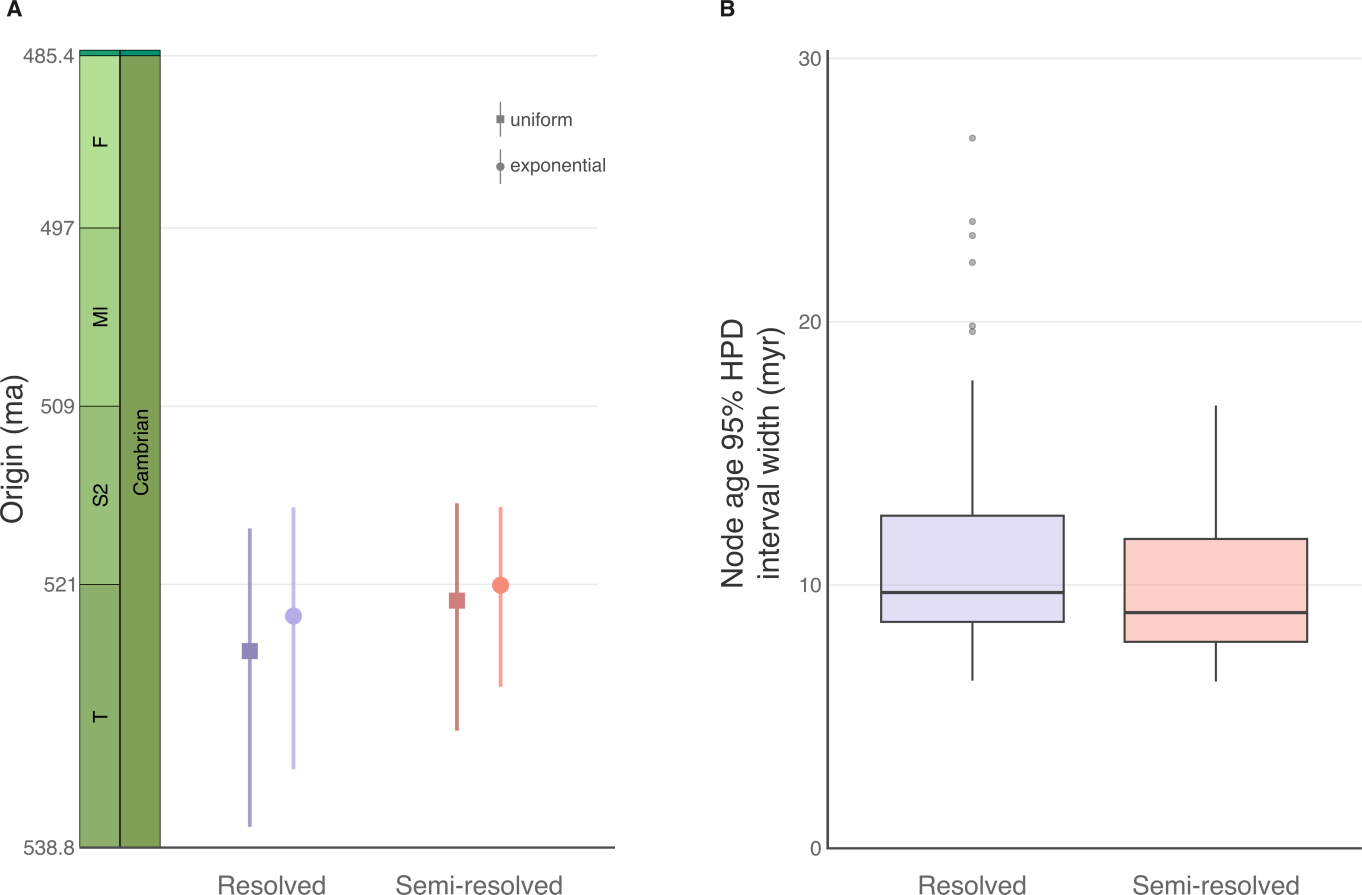
Comparison of effects of resolved and semi-resolved FBD analyses on inferred origin and divergence times. (A) Ninety-five per cent highest posterior densities (HPDs) and medians of the origin time for each type of analysis and prior distribution (square = uniform; circle = exponential). (B) Ninety-five per cent HPD width for all nodes in each MCC tree.

Estimates of the origin age were more precise with the semi-resolved analysis than with the resolved analysis (figure 3A). The 95% highest posterior density (HPD) interval for the origin age in the semi-resolved analysis was 25.7% narrower with a uniform prior and 31.3% narrower with an exponential prior than the resolved analysis. The HPDs were almost entirely overlapping, demonstrating the semi-resolved analysis mainly produced a marked decrease in the uncertainty. Notably, however, the median inferred ages were slightly younger for the semi-resolved analyses.

Inferred clade divergence times were also markedly more precise for the semi-resolved analysis than the resolved analysis (figure 3B). The largest uncertainty interval around a node for the resolved analysis was 27 myr, while it was 17 myr for the semi-resolved analysis.

## 4. Discussion

Our comparative analyses show that a combined analysis including both fossils with and without character data using taxonomic constraints is not only possible in practice but also outperforms analyses that include only taxa scored into a morphological matrix for fossil groups. As expected, more fossil data and a more complete representation of their temporal distribution (as in the ‘semi-resolved’ analysis) produce markedly more precise parameter estimates. Any analyses where divergence time estimation is of interest would therefore benefit from the combined ‘semi-resolved’ approach. Also unsurprisingly, trees produced from the ‘semi-resolved’ analysis show much higher levels of stratigraphic congruence than those from the ‘resolved’ analysis. That is, they better fit the order of appearance of taxa in the fossil record. This may be an intuitive result, but it illustrates how sampling only taxa with abundant morphological characters has the potential to generate misleading results.

Stratigraphic age information for taxa with morphology has previously been shown to improve phylogenetic analyses of those taxa [10,14]. In such analyses, age information causes some regions of treespace to become implausible. Moreover, topological changes to regions of the tree with weaker character signal can be induced by age information [38]. A crude way to visualize these changes is by simply comparing the MCC trees and the ghost ranges (electronic supplementary material, figure S7). However, MCC trees are often unreliable for tree distributions with high levels of uncertainty, as is the case for most morphological datasets [39]. Understanding and comparing the posterior distributions is therefore aided here by the visualization of treespace, a powerful tool for exploring distributions of trees [40], but underutilized particularly for fossil groups [41]. In this treespace, trees from the ‘semi-resolved’ analysis did not occupy the more stratigraphically incongruent regions and clustered more densely around the regions of highest stratigraphic congruence. Our results therefore show that age information of taxa other than just those with morphology can further cause stratigraphically incongruent regions of treespace to become implausible.

The ‘semi-resolved’ analysis had a significant and substantial effect on the SCI, but had a larger effect on the MIG and GER metrics. The SCI is a proportion of stratigraphically consistent nodes in a tree and does not consider the length of ghost ranges, like the MIG and GER do [31]. This suggests that the restructuring of only a few nodes (or taxa) to stratigraphically consistent positions can break up long branches. This is a direct consequence of a more representative distribution of fossil sampling ages.

Leaf stability is another important aspect of a distribution of topologies. Taxa whose phylogenetic position are particularly unstable throughout a distribution of trees—‘rogues’—are problematic as they obscure otherwise stable phylogenetic relationships [19,37,42]. They also obliterate node support values and the ability to resolve relationships in consensus trees. Here, the ‘semi-resolved’ analysis produced fewer rogue taxa. It may be that the additional constraints provided by the stratigraphic information prevent would-be rogues from attaching to very distant, stratigraphically inconsistent, branches. This would also align with the aforementioned observation that age information exerts the strongest effects in sections of the tree with weaker character signal [38]. Nevertheless, this higher leaf stability affords more confidence in the inferred clade relationships than the ‘resolved’ analysis.

Perhaps due to fewer rogue taxa obscuring the resolution of deeper nodes, the semi-resolved analysis produced a majority rule consensus tree with higher information content than the resolved analysis. Splits in a summary tree are not all equally informative, for example, a split leading to two species in the same genus is less informative than a split separating two families. While intuitive, formally, this is because the probability of larger clades and splits among them is much lower in a posterior distribution. When a consensus tree captures relationships of lower probability in the tree distribution it is therefore more informative [37,43,44]. Consequently, majority rule consensus trees with the same number of resolved splits may not necessarily be equally informative. Here, the higher information content in the semi-resolved majority rule consensus tree is a consequence of it capturing larger clade relationships at deeper nodes (electronic supplementary material, figure S4). This demonstrates that the distribution of trees is narrower, and the consensus tree can communicate the captured relationships with less uncertainty. Put another way, this reflects a higher level of clade stability in the posterior distribution.

The combined approach to incorporating fossils into FBD analyses, here termed ‘semi-resolved’, offers an intuitive and relatively simple means of mitigating taxon sampling biases, while providing a more comprehensive utilization of the fossil record. Yet, this strategy has historically been overlooked. We tested and demonstrated the effects of this approach by simply incorporating bulk occurrence stratigraphic age information from the PBDB into FBD phylogenetic analyses of a fossil group. This information is readily available and easily obtained, so may offer a high benefit : effort ratio.

Desirable properties notwithstanding, occurrences must be carefully scrutinized and assigned to taxonomic constraints with caution. A cleaning procedure such as that presented here should always be performed on the dataset before inclusion in any analyses. The amount and type of cleaning will always depend on the taxon of interest and the individual dataset, however (see Jones *et al*. [45] for a full discussion on cleaning occurrence datasets and [5] for a discussion on preparing PBDB data for analysis using the FBD model). Barido‐Sottani *et al*. [10] also show that erroneously placed fossil taxa can not only nullify the positive effects but reduce overall accuracy in phylogenetic inference. Appropriately assigning taxonomic constraints to a dataset/analysis therefore requires a taxonomic expert familiar with up-to-date taxonomy for the group of interest. It may also sometimes be difficult to detect taxonomic errors in a dataset; however, when taxonomic uncertainty is higher, one may utilize more relaxed constraints, e.g. family instead of genus. This strategy mitigates the negative effects of incorrect taxonomic placement, while still offering much of the benefit of the fossils’ inclusion [46]. In some cases, though, it may be that occurrence datasets obtained from the PBDB for a given group may not be appropriate for certain analyses. In light of this, while the results we present here are encouraging, we echo sentiments in Barido‐Sottani *et al*. [10] that developments in phylogenetic and palaeobiological models are no replacement for fundamental taxonomic, stratigraphic and systematic research.

Here, we corroborate previous simulation results [10] with a complex empirical scenario and further emphasize the utility of stratigraphic age information in phylogenetic analyses. While the focus here was on a group of extinct organisms, our findings would also be applicable to groups including extant taxa. We therefore encourage researchers to consider the full amount of fossil information that fundamental taxonomic and stratigraphic work has afforded.

## Data Availability

The morphological matrix, associated data and Beast2 XML configuration files are publicly available from MorphoBank (Project 5740) [26]. All additional age data and code for all analyses are included as electronic supplementary material. Supplementary material is available online [47].
